# Serum anti-NMDA receptor antibodies are linked to memory impairment 12 months after stroke

**DOI:** 10.1038/s41380-024-02744-w

**Published:** 2024-10-30

**Authors:** Friederike A. Arlt, Pia S. Sperber, Regina von Rennenberg, Pimrapat Gebert, Bianca Teegen, Marios K. Georgakis, Rong Fang, Anna Dewenter, Michael Görtler, Gabor C. Petzold, Silke Wunderlich, Inga Zerr, Martin Dichgans, Harald Prüss, Matthias Endres, On behalf of the DEMDAS Investigators, Rong Fang, Anna Dewenter, Michael Görtler, Gabor C. Petzold, Silke Wunderlich, Inga Zerr, Martin Dichgans, Harald Prüss, Matthias Endres, Thomas Liman, Christian Nolte, Lucia Kerti, Tatjana Wittenberg, Jan F. Scheitz, Pia S. Sperber, Alexander H. Nave, Anna Ibaroule Kufner, Felix Bode, Sebastian Stösser, Julius N. Meißner, Taraneh Ebrahimi, Julia Nordsiek, Niklas Beckonert, Peter Hermann, Matthias Schmitz, Stefan Goebel, Julia Schütte-Schmidt, Sabine Nuhn, Corinna Volpers, Peter Dechent, Matthias Bähr, Wenzel Glanz, Marios Georgakis, Steffen Tiedt, Karin Waegemann, Daniel Janowitz, Benno Ikenberg, Kathleen Bermkopf, Christiane Huber, Michael Wagner, Katja Neumann, Annika Spottke, Tony Stöcker, Marco Dühring, Oliver Speck, Emrah Duezel, Peter Bartenstein

**Affiliations:** 1 Department of Neurology and Experimental Neurology, Charité-Universitätsmedizin Berlin, corporate member of Freie Universität and Humboldt-Universität zu Berlin, Berlin, Germany; 2 German Center for Neurodegenerative Diseases (DZNE) Berlin, Berlin, Germany; 3 Experimental and Clinical Research Center, a cooperation between Max Delbrück Center for Molecular Medicine in the Helmholtz Association and Charité-Universitätsmedizin Berlin, Corporate Member of Freie Universität Berlin and Humboldt-Universität zu Berlin, Berlin, Germany; 4 NeuroCure Clinical Research Center, Charité-Universitätsmedizin Berlin, Corporate Member of Freie Universität and Humboldt-Universität zu Berlin, Berlin, Germany; 5 German Center for Cardiovascular Diseases (DZHK), Partner Site Berlin, Berlin, Germany; 6 Institute of Biometry and Clinical Epidemiology, Charité-Universitätsmedizin Berlin, Corporate member of Freie Universität and Humboldt-Universität zu Berlin, Berlin, Germany; 7 Clinical Immunological Laboratory Prof. Stöcker, Groß Grönau, Germany; 8 Institute for Stroke and Dementia Research (ISD), University Hospital, LMU Munich, Munich, Germany; 9 Department of Neurology, University Hospital, Otto-von-Guericke University Magdeburg, Magdeburg, Germany; 10 German Center for Neurodegenerative Diseases (DZNE) Magdeburg, Magdeburg, Germany; 11 Department of Vascular Neurology, University Hospital Bonn, Bonn, Germany; 12 German Center for Neurodegenerative Diseases (DZNE) Bonn, Bonn, Germany; 13 Department of Neurology, Klinikum rechts der Isar School of Medicine, Technical University of Munich, Munich, Germany; 14 Department of Neurology, University Medical Center Göttingen, Göttingen, Germany; 15 German Center for Neurodegenerative Diseases (DZNE) Göttingen, Göttingen, Germany; 16 German Center for Neurodegenerative Diseases (DZNE) Munich, Munich, Germany; 17 Munich Cluster for Systems Neurology (SyNergy), Munich, Germany; 18 German Center for Cardiovascular Research (DZHK, Munich), Munich, Germany; 19 Center for Stroke Research Berlin (CSB), Charité-Universitätsmedizin Berlin, Corporate Member of Freie Universität and Humboldt-Universität zu Berlin, Berlin, Germany; 20 German Center for Mental Health (DZPG), Partner Site Berlin, Berlin, Germany; 21 Department of Neurology, Carl Von Ossietzky University, Oldenburg, Germany; 22 Berlin Institute of Health (BIH), Berlin, Germany; 23 Cluster of Excellence Nanoscale Microscopy and Molecular Physiology of the Brain (CNMPB), Göttingen, Germany; 24 Department of Neurodegenerative Diseases and Geriatric Psychiatry, University Hospital Bonn, Bonn, Germany; 25 Medical Image Analysis Center (MIAC AG) and qbig, Department of Biomedical Engineering, University of Basel, Basel, Switzerland; 26 Department of Biomedical Magnetic Resonance, Institute for Physics, Otto-von-Guericke University Magdeburg, Magdeburg, Germany; 27 Leibniz Institute for Neurobiology, Magdeburg, Germany; 28 Center for Behavioral Brain Sciences, Magdeburg, Germany; 29 Department of Nuclear Medicine, University Hospital, LMU Munich, Munich, Germany

**Keywords:** Prognostic markers, Neuroscience, Psychiatric disorders

## Abstract

Patients suffering from strokes are at increased risk of developing post-stroke dementia. Serum anti-NMDA receptor autoantibodies (NMDAR1-abs) have been associated with unfavorable post-stroke outcomes. However, their effect on specific cognitive domains remains unclear. We used data from the prospective multicenter DZNE—mechanisms after stroke (DEMDAS) cohort, and measured NMDAR1-abs in serum at baseline. Cognitive function was assessed with a comprehensive neuropsychological test battery at 6- and 12-months follow-up. We employed crude and stepwise confounder adjusted linear and logistic regression models as well as generalized estimating equation models (GEE) to determine the relevance of NMDAR1-abs seropositivity on cognitive function after stroke. 10.2% (58/569) DEMDAS patients were NMDAR1-abs seropositive (IgM:n = 44/IgA:n = 21/IgG:n = 2). Seropositivity was not associated with global cognitive impairment after stroke. However, NMDAR1-abs seropositive patients performed lower in the memory domain (β_adjusted_ = −0.11; 95%CI = −0.57 to −0.03) and were at increased risk for memory impairment (OR_adjusted _= 3.8; 95%CI = 1.33–10.82) compared to seronegative patients, 12 months after stroke. Further, NMDAR1-abs were linked to memory impairment over time in GEE from 6- to 12-months follow-up (OR_adjusted _= 2.41; 95%CI = 1.05–5.49). Our data suggests that NMDAR1-abs contribute to memory dysfunction 1 year after stroke while not affecting other cognitive subdomains. Hence, antineuronal autoimmunity may be involved in distinct mechanisms of post-stroke memory impairment. *Clinical trial name and registration number*: The Determinants of Dementia After Stroke (DEMDAS; study identifier on clinical trials.gov: NCT01334749)

## Introduction

A stroke is a devastating event with far-reaching consequences for further life of the affected person. Despite the development of highly effective treatments in recent decades, the global impact of stroke on individuals, care takers, and economy remains enormous [1]. While modern treatments have significantly reduced death and physical disability after stroke, the burden of cognitive impairment increases with increased survival and aging [2]. A deterioration of cognitive abilities is commonly observed after stroke, including after minor events and even after transient ischemic attacks [3]. However, the precise mechanisms that lead to cognitive impairment are not known. Hence, effective prevention and treatment strategies are lacking [4]. Serum anti-NMDA-receptor GluN1 (NR1) autoantibodies (NMDAR1-abs), primarily of immunoglobulin (Ig) A and IgM isotype, have been observed in presumably healthy individuals and in patients with various diseases [5–8]. While previously thought to have beneficial effects on ischemic brain lesion evolution in stroke [9, 10], they have been associated with unfavorable post-stroke clinical outcomes, including cognitive outcomes [7, 11, 12]. Recent work suggests impaired neuropsychiatric outcomes after stroke, including cognitive dysfunction, particularly in those with high titers as assessed by screening tests [12, 13]. Interestingly, similar findings have been reported in NMDAR1-abs seropositive patients with other diseases (e.g., cancer patients), suggesting a cross-disease pathophysiological relevance of these antibodies [14–16]. It was suggested previously that serum NMDAR1-abs may enter the brain after blood-brain barrier disruption and exert pathological effects [17, 18], which may explain cognitive dysfunction after stroke. However, detailed and differentiated neuropsychiatric data elucidating a potential link between NMDAR1-abs seropositivity and cognitive impairment for stroke outcome are not available. In the present study, we estimated the impact of NMDAR1-abs seropositivity on global cognitive function and various subdomains of cognitive function using high-quality prospective data from the Determinants of Dementia After Stroke (DEMDAS) study.

## Subjects and methods

### Study design, registrations, and patient population

DEMDAS (study identifier on clinical trials.gov: NCT01334749) is an ongoing national multicenter, prospective observational study with the aim to investigate mechanisms leading to cognitive dysfunction and dementia after stroke. Participating centers are listed in Supplementary (Suppl.) Table 1. Within the study, 600 patients with ischemic or hemorrhagic stroke are followed over time in person at seven different sites in Germany. A detailed protocol of the study design has been published previously [19]. In brief, patients aged at least 18 years with stroke defined by a new neurological deficit within the previous 5 days and a new lesion on magnet resonance imaging (MRI) or computer tomography are included. Patients with prior dementia, defined as >64 sum points in the short version of the ‘Informant Questionnaire on Cognitive Decline in the Elderly’ (IQCODE) [20], and patients with a life expectancy of less than 3 years due to malignancy were excluded. The total follow-up duration for study completion is planned for 5 years. For this investigation, data from the 6- and 12-months follow-up (FU) visits were used. A detailed characterization of study participants was performed at baseline.

### Antibody measurements

Blood serum samples were taken from each participant at study inclusion and stored at −80 °C before first-time ever thawing for antibody measurement. NMDAR1-abs were measured at the Clinical Immunological Laboratory Prof. Stöcker using reagents of the EUROIMMUN AG with fixed cell-based assays using *GluN1* transfected Human Embryonic Kidney 239 cells, and indirect immunofluorescence as previously described in detail [21]. We measured IgM, IgA, and IgG isotype NMDAR1-abs, and any titer above or equal 1:10 was considered seropositive. Titer dilution steps were 1:10, 1:32, 1:100, 1:320, and 1:1000.

### Assessment of cognitive function and definition of outcome parameters

Cognitive abilities in five different domains (language, memory, visuospatial function, executive function, and attention) were assessed by a comprehensive neuropsychological testing. Mainly, the Consortium to Establish a Registry for Alzheimer’s Disease Plus (CERAD-Plus) battery in addition to other tests were used, in line with a previous work [22, 23]. CERAD-Plus includes a test for language-specific function, “Semantic and Phonemic Fluency” and “Boston Naming” [22], and additionally we used the language items from Mini Mental State Examination (MMSE) [24]. Furthermore, to examine memory function, CERAD-Plus includes Word List Learning/Recall, Recognition, and Figure Recall. Immediate and delayed recall was tested by the Rey-Osterrieth Complex Figure (ROCF) [25]. To examine visuospatial function, CERAD-Plus includes the Figure Drawing Test and we applied additionally the copy test of ROCF [22, 25]. For executive function, CERAD-Plus uses the Trail Making Test Part B and the Stroop-Colour-Word-Interference Test [26]. We tested attention with the Trail Making Test Part A from CERAD-Plus and additionally with the Digit-Symbol-Substitution Test of the Wechsler Intelligence Scale [27]. Age, sex, and education standardized z-scores were calculated from a reference normative population, as previously described [23]. Next, a domain-specific z-score was averaged for each domain and subsequently, these were calculated into a global cognitive score as previously described, defining the global cognitive function in our study [23]. Performance of less than −1.5 z-score points was used to define impairment in any cognitive domain or in global cognitive function, respectively [28]. At baseline, cognitive impairment was assessed using Montreal Cognitive Assessment (MoCA) and (if MoCA was unavailable) MMSE scores [29]. Impairment was defined as <26 points in MoCA, or <24 in MMSE [30, 31].

### Neuroimaging

Cranial MRI scans (3 Tesla, Siemens Healthineers, Erlangen, Germany) were conducted at baseline. The MRI protocol included 3D T1-weighted magnetization prepared rapid gradient echo (MPRAGE), 3D fluid-attenuated inversion recovery (FLAIR), diffusion-weighted imaging (DWI) with various diffusion directions, T2-weighted turbo spin echo, and T2*-weighted fast low angle shot (FLASH) gradient echo. Intracranial volumes, total brain volumes, and stroke lesion size were derived as previously described [23]. Hippocampal volumes were assessed using the FreeSurfer software (version 5.3; http://surfer.nmr.mgh.harvard.edu/). Normalized brain volumes were adjusted to total intracranial volumes to account for differences in head size (normalized brain volume = [total brain volume + infract volume]/total intracranial volume). Hippocampal volumes were likewise adjusted to intracranial volumes (normalized hippocampal volume = [right hippocampal volume + left hippocampal volume]/total intracranial volume). Hippocampal volumes were calculated for patients without acute stroke lesions in the hippocampal area.

### Statistics

Continuous data is presented as mean with standard deviation (SD) and median along with interquartile range (25th and 75th percentile), while categorical variables are displayed as absolute counts (N) and corresponding percentages (%). We calculated absolute standardized mean differences to evaluate how well the groups of NMDAR1-abs seropositive and seronegative patients are balanced regarding baseline characteristics [32]. To estimate effects of NMDAR1-abs serostatus on global and domain-specific test performance at 6- and 12-months FU, we used linear regression models. Regarding the binary cognitive outcomes at both timepoints, we used logistic regression models. We calculated beta effects and odds ratios (OR) with corresponding 95% confidence intervals (95%CI), respectively. To estimate the effects of NMDAR1-abs seropositivity on cognitive outcome over two timepoints, i.e., 6 to 12 months after stroke, we calculated ORs from generalized estimating equation (GEE) models, comparing seropositive vs. seronegative participants, while encountering the dependency of observations (within subject measurements). To adjust for confounding factors, we conducted a stepwise adjustment: the first model was to explore the unadjusted association (crude, model 1), a partially adjusted model was built with age (continuous), sex (dichotomous), and education (in school educational years, continuous) (model 2), and a fully adjusted model incorporating a propensity score (model 3). The propensity score was calculated from logistic regression models including age (continuous), sex (dichotomous), education (continuous), ever smoking (dichotomous), habitual alcohol consumption (dichotomous), severe chronic disease leading to retirement, obligate support in daily life, or manifest reduction of life quality (dichotomous), previous stroke or transitory ischemic attack (dichotomous), cardiovascular diseases (dichotomous), and other organic brain diseases including other cerebrovascular events excluding stroke or transitory ischemic attack (dichotomous), with NMDAR1-abs serostatus as dependent variable. All confounders were considered to have a potential impact on NMDAR1-abs serostatus at time of assessment and on the respective cognitive outcome.

We additionally investigated post-hoc whether our observed results may be influenced by other neuropsychiatric sequelae: To measure the severity of depressive symptoms, we used the 20-item center for epidemiological studies depression (CES-D) scale, which has been validated for use in German and in stroke patients [33, 34]. To measure fatigue, we used the first 20 items (excluding the visual analog scales, i.e., item 21–24) of the Fatigue Assessment Questionnaire (FAQ), which was also validated for the use in stroke patients [35, 36]. We visually inspected the linear relationship of these measures with z-scores from the memory domain at 6- and 12-month post-stroke and calculated correlation coefficients. Additionally, we investigated whether the observed effects on the memory domain may be explained by hippocampal volume at baseline. Therefore, we correlated baseline hippocampal stroke volumes with the z-scores of the memory domain at 6- and 12-month post-stroke.

Data preparation and statistical analyses were conducted using SPSS Statistics 28.0.1.0 (IBM, Armonk, NY, USA). Data visualization was conducted using R version 4.2.3 with the ggplot2 package and Prism Version 9.4.1 (GraphPad Software, San Diego, CA, USA).

### Ethics and standard protocol approvals

The DEMDAS study was conducted in accordance with the guidelines of the Declaration of Helsinki and was approved by the local ethics committees of the participating centers. All participants gave written informed consent to participate in the study and to the analysis of serum biomarkers. Our reporting follows the Strengthening the Reporting of Observational Studies in Epidemiology (STROBE) guidelines [37].

## Results

### Patient population

**Fig. 1:**
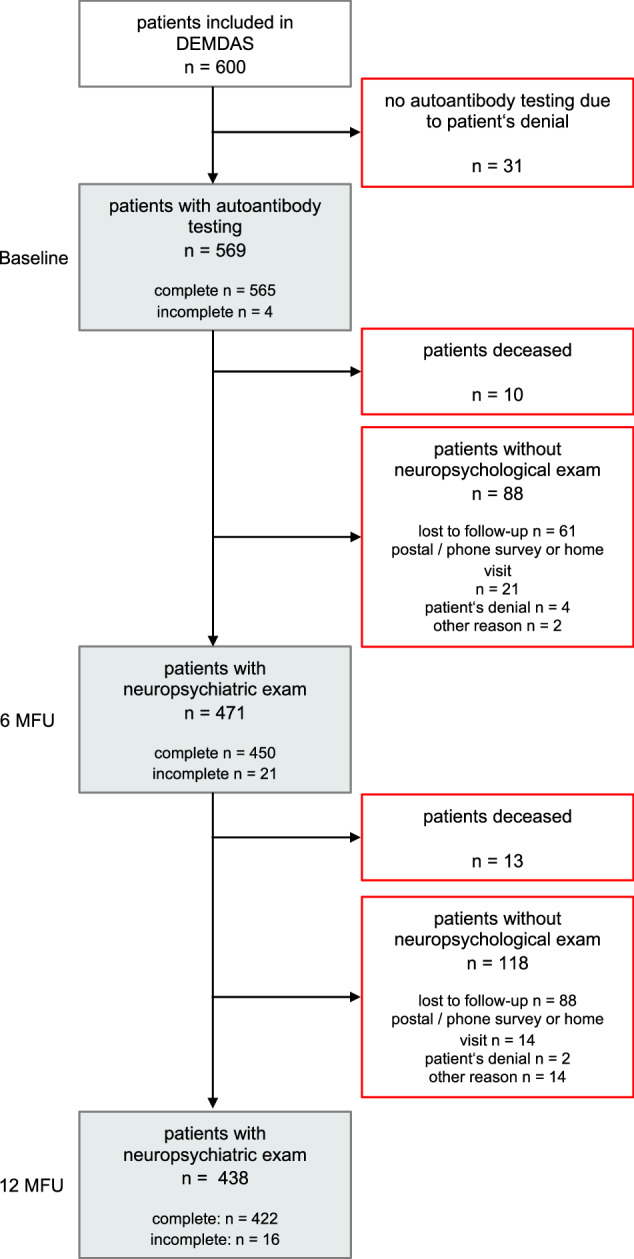
Flowchart of patient inclusion and exclusion. Gray boxes indicate that participants were included in the analysis, while red boxes represent participants that were excluded from the analysis. Autoantibody indicates anti-NMDAR1 (GluN1) autoantibodies. MFU months follow-up.

Between January 2014 and January 2019, DEMDAS recruited a total of 600 patients, of whom 569 patients were tested for any serum NMDAR1-abs and were thus eligible for our analysis. The median day of blood sampling after index stroke was 1 (IQR: 1–2). The flowchart depicted in Fig. 1 describes the inclusion and exclusion of patients and provides an overview of the number of neuropsychological examinations after 6 and 12 months. The most common reason for omission of serological antibody testing was patient refusal to have blood drawn, and missing complete autoantibody testing in four patients was due to limited serum amounts. The lack of a complete neuropsychological examination was mainly due to lost follow-ups, patients’ inability to complete the examination for health reasons, or because the patient preferred to be followed by mail or telephone, which did not include comprehensive neuropsychological testing. Overall, 450 patients at 6 months (80.7% of seronegatives vs. 63.8% of seropositives) and 423 patients at 12 months (75.5% of seronegatives vs. 62.1% of seropositives) received a complete neuropsychological assessment that allowed calculation of a global cognitive score. An incomplete cognitive assessment testing at least one cognitive domain was obtained in 471 patients at 6-months FU and 438 patients at 12-months FU, respectively. A total of 13 patients died (2 seropositive and 11 seronegative), corresponding to 2.3% of the missing data.

### Baseline characteristics

Baseline characteristics of the original study group (n = 569) are shown in Table 1, stratified by NMDAR1-abs serostatus, and with corresponding absolute standardized mean differences. Overall, the cohort included 66.4% men and had predominantly ischemic strokes (97.4%). The majority of patients had mild to moderate strokes with a median NIHSS score of 3 (IQR: 1–5). At baseline autoantibody testing, 58 patients (10.2%) were seropositive for any form of NMDAR1-abs, with IgM (n = 44) and IgA (n = 21) predominating and only 2 patients having NMDAR1-abs of the IgG isotype. Seropositive patients were generally older with a median age of 74 (IQR: 67–80) vs. 68 years (IQR: 60–75). In addition, seropositive patients had more cardiovascular risk factors, including a history of previous ischemic cerebrovascular events (19.0% vs. 12.3%). Imaging data showed similar stroke lesions size in both groups (mean of 0.20% in seropositive vs. 0.15% in seronegative patients). However, hippocampal volumes and total brain volumes (normalized to intracranial volumes) were slightly smaller in seropositive patients (4.3‰ vs. 4.7‰; mean difference: 0.4; 95%CI = 0.2 to 0.6 and 66% vs. 68%; mean difference: 2.4; 95%CI = 0.76 to 3.98). Overall, 24.4% of patients received intravenous thrombolysis therapy, with more seronegative than more seropositive patients (25.0% vs. 17.2%). Cognitive impairment at baseline assessed with bedside screening tests (i.e., <26 points in MoCA, and <24 in MMSE) was noted in 53.4% of the total cohort, without major differences between seropositive and seronegative patients (52.9% of seronegative vs. 58.2% of seropositive patients). Apolipoprotein E genotyping and history of depression at baseline also revealed no differences between seronegative and seropositive patients.

**Table 1:** Baseline characteristics of DEMDAS patients stratified upon anti-NMDAR1 autoantibody serostatus.

Baseline characteristics	Total	Seronegative	Seropositive	ASMD
DEMDAS participants, anti-neuronal antibody testing, n (%)	569 (100)	507 (89.1)	58 (10.2)	
Blood sampling days after index stroke, median (IQR)	1 (1–2)	1 (1–2)	2 (1–2)	0.110
Demographic variables				
Age in years, median (IQR)	69 (60–76)	68 (60–75)	74 (67–80)	0.528
Male sex, n (%)	378 (66.4)	335 (66.1)	42 (72.4)	0.138
Years of education, median (IQR)	13 (12–16)	13 (12–16)	13 (12–17)	0.056
Cardiovascular risk factors and previously existing diseases				
Systolic blood pressure in mmHg, median (IQR)	139 (128–151)	139 (129–151)	139 (122–149)	0.141
Diastolic blood pressure in mmHg, median (IQR)	80 (70–89)	80 (70–89)	79 (70–85)	0.287
Body mass index in kg/m^2^, median (IQR)	26.6 (24.3–29.2)	26.5 (24.2–29.2)	27.5 (24.6–29.4)	0.018
Habitual alcohol consumption, n (%)	419 (73.9)	375 (74.3)	41 (70.7)	0.116
Ever smoker, n (%)	342 (60.1)	307 (60.6)	31 (53.4)	0.144
History of hypertension, n (%)	318 (55.9)	282 (57.0)	35 (62.5)	0.113
History of diabetes, n (%)	84 (14.8)	72 (14.2)	12 (20.7)	0.171
History of any hyper-/ dyslipidemia, n (%)	165 (29.7)	141 (28.5)	23 (39.7)	0.236
History of peripheral artery disease, n (%)	16 (2.8)	13 (2.6)	3 (5.3)	0.139
History of coronary artery disease, n (%)	32 (5.6)	25 (5.0)	7 (12.1)	0.257
History of myocardial ischemia, n (%)	31 (5.4)	26 (5.1)	5 (8.6)	0.138
History of angina pectoris, n (%)	14 (2.5)	13 (2.6)	1 (1.8)	0.057
History of atrial fibrillation, n (%)	63 (11.2)	55 (11.0)	8 (14.3)	0.100
History of any cardiovascular disease, n (%)	392 (70.0)	347 (69.4)	44 (77.2)	0.177
Previous stroke or TIA, n (%)	74 (13.1)	62 (12.3)	11 (19.0)	0.185
History of other organic brain disease^a^, n (%)	18 (3.2)	15 (3.0)	3 (5.2)	0.070
History of severe disease^b^, n (%)	68 (12.0)	58 (11.5)	9 (15.5)	0.119
History of depression, n (%)	35 (6.1)	33 (6.5)	2 (3.4)	0.209
*APOE* genotype				0.195
0 ε4 allele, n (%)	383 (78.6)	346 (78.6)	35 (81.4)	
1 ε4 allele, n (%)	96 (19.7)	86 (19.5)	8 (18.6)	
2 ε4 alleles, n (%)	8 (1.6)	8 (1.8)	0 (0.0)	
Index stroke classification				0.070
Ischemic stroke, n (%)	554 (97.4)	493 (97.2)	57 (98.3)	
Hemorrhagic stroke, n (%)	15 (2.6)	14 (2.8)	1 (1.7)	
TOAST, n (%)				0.276
Large artery atherosclerosis	155 (28.0)	134 (27.2)	19 (33.4)	
Cardio embolism	126 (22.7)	113 (22.8)	13 (22.8)	
Small vessel disease	66 (11.9)	59 (12)	7 (12.3)	
Dissection	20 (3.6)	19 (3.9)	1 (1.8)	
Other etiology^c^	42 (7.6)	56 (11.4)	5 (8.8)	
Undetermined etiology	105 (18.8)	96 (19.5)	7 (12.3)	
Diagnostic workup incomplete	41 (7.4)	35 (7.1)	6 (10.5)	
Intravenous thrombolysis, n (%)	139 (24.4)	127 (25.0)	10 (17.2)	0.224
MRI variables				
Stroke lesion volume in mm^3^, median (IQR)	2248 (528–12,652)	2144 (528–13,136)	2804 (402–9936)	0.073
Normalized stroke lesion volume^d^ in %, median (IQR)	0.15 (0.03–0.78)	0.15 (0.03–0.78)	0.20 (0.03–0.61)	0.078
Total brain volume^e^ in mm^3^, median IQR	1,042,817 (953,040–1,138,041)	1,048,148 (957,922–1,143,259)	1,015,360 (936,025–1,094,772)	0.324
Normalized brain volume^d^ in %, mean SD	68 (5.4)	68 (5.4)	66 (5.2)	0.464
Total hippocampal volume (left + right) in mm^3^, median IQR	7203 (6566–7955)	7252 (6684–7981)	6617 (5742–7430)	0.510
Normalized hippocampal volume^d^ in ‰, mean SD	4.7 (0.6)	4.7 (0.6)	4.3 (0.7)	0.505
Hippocampal stroke, n (%)	41 (7.7)	38 (8.0)	3 (5.7)	0.093
White matter lesions^f^, n (%)	349 (61.4)	307 (60.7)	38 (65.5)	0.190
Chronic stroke lesions, n (%)	161 (29)	144 (29.1)	15 (26.8)	0.051
Clinical/cognitive assessment				
NIHSS, median (IQR)	3 (1–5)	2 (1–5)	3 (1–5)	0.139
Baseline MoCA, median (IQR)	25 (22–27)	25 (22–27)	24 (22–27)	0.286
Cognitive impairment at baseline^g^, n (%)	291 (53.4)	257 (52.9)	32 (58.2)	0.107
IQCODE score, median (IQR)	48 (48–50)	48 (48–49)	48 (48–50)	0.052
Baseline mRS, median (IQR)	2 (1–3)	2 (1–3)	2 (1–2)	0.005

Due to rounding, values might not add to 100%. Missing values were <10% except for *APOE* Genotyping, (*n* = 487), Baseline MoCA (*n* = 505).

*ASMD* absolute standardized mean difference, *TIA* transitory ischemic attack, *APOE* Apolipoprotein E, *NIHSS* National Institutes of Health Scale, *MMSE* Mini Mental State Examination, *MoCA* Montreal Cognitive Assessment, *IQCODE* Informant Questionnaire on Cognitive Decline in the Elderly, *mRS* modified ranking scale.

^a^including other cerebrovascular events (excluding ischemic stroke and transitory ischemic attack).

^b^severe disease that led to retirement or obligate support in daily life or manifest reduction of quality of life.

^c^other defined causes, and several potential causes.

^d^divided by total intracranial volume.

^e^brain volume + infarct volume.

^g^any white matter lesions: punctual, early confluent, wide confluent.

^g^MoCA < 26 or MMSE < 24 if MoCA was not available (*n* = 64).

### NMDAR1-abs and cognitive outcome—descriptive data

**Fig. 2:**
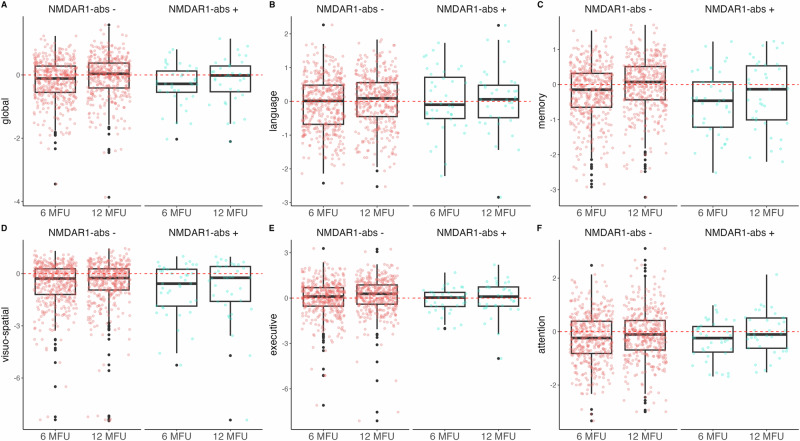
Global and subdomain scoring from the Consortium to Establish a Registry for Alzheimer’s Disease Plus’–battery (CERAD-Plus) from anti-NMDAR1 autoantibody seronegative and seropositive patients. Red dots represent single participants’ z-scores of anti-NMDAR1 autoantibody seronegative patients while blue dots display single participants’ z-scores of anti-NMDAR1 autoantibody seropositive patients, with a boxplot overlay and emphasized zero-line (red dashed line). **A** Global cognitive test performance. **B** Test performance in the language domain. **C** Test performance in the memory domain. **D** Test performance in the visuo-spatial domain. **E** Test performance in the executive domain. **F** Test performance in the attention domain. Age, sex, and education standardized z-scores were calculated from a reference normative population. MFU months follow-up.

Overall, cognitive test performance (global and domain specific) improved from 6 to 12 months after stroke as shown in Fig. 2. Z-scores for global cognitive function improved in both groups with similar total score point differences (Δ = 0.13 score points in seronegative and Δ = 0.14 in seropositive patients, Fig. 2A). However, seropositive patients started with lower global z-scores at 6 months (mean of −0.31 [SD = 0.64] vs. −0.18 [SD = 0.65], Fig. 2A) thereby also reaching a lower mean global z-score at 12-months FU compared to seronegative patients (mean of −0.17 [SD = 0.72] vs. −0.05 [SD = 0.67]). Likewise, relative amounts of patients with global cognitive impairment were different at 6-months FU with 18/409 (4.4%) seronegative compared to 3/37 (8.1%) seropositive patients. Moreover, at 12-months FU, global cognitive impairment decreased in seronegative to only 11/371 (2.9%) while 3/37 (8.1%) seropositive patients remained impaired (Suppl. Table 2). In cognitive test performance of the memory domain, NMDAR1-abs seropositive patients showed also lower z-scores at both timepoints with similar total score point differences (Fig. 2C). In line, relative amounts of memory impairment were higher in seropositive compared to seronegative patients at 6-months FU (6/40 (15%) vs. 32/425 (7.5%), Suppl. Table 2). Similar to global cognitive impairment, seronegative patients showed less memory impairment with only 15/396 (3.8%), while 6/38 (15.8%) seropositive patients remained impaired after 12 months (Suppl. Table 2). The observed differences in memory performance at the 12-month FU between seropositive and seronegative patients appeared to be mainly driven by those with IgA antibodies. In this subgroup of seropositive patients, z-scores showed only minimal improvement from the 6- to the 12-month FU, in contrast to patients with IgM antibodies (Suppl. Fig. 1C). Z-scores and counts of cognitively impaired individuals in other cognitive domains are shown in Fig. 2B, D, E, F, Suppl. Fig. 1, and Suppl. Table 2.

### NMDAR1-abs and cognitive outcome—inferential analyses

**Fig. 3:**
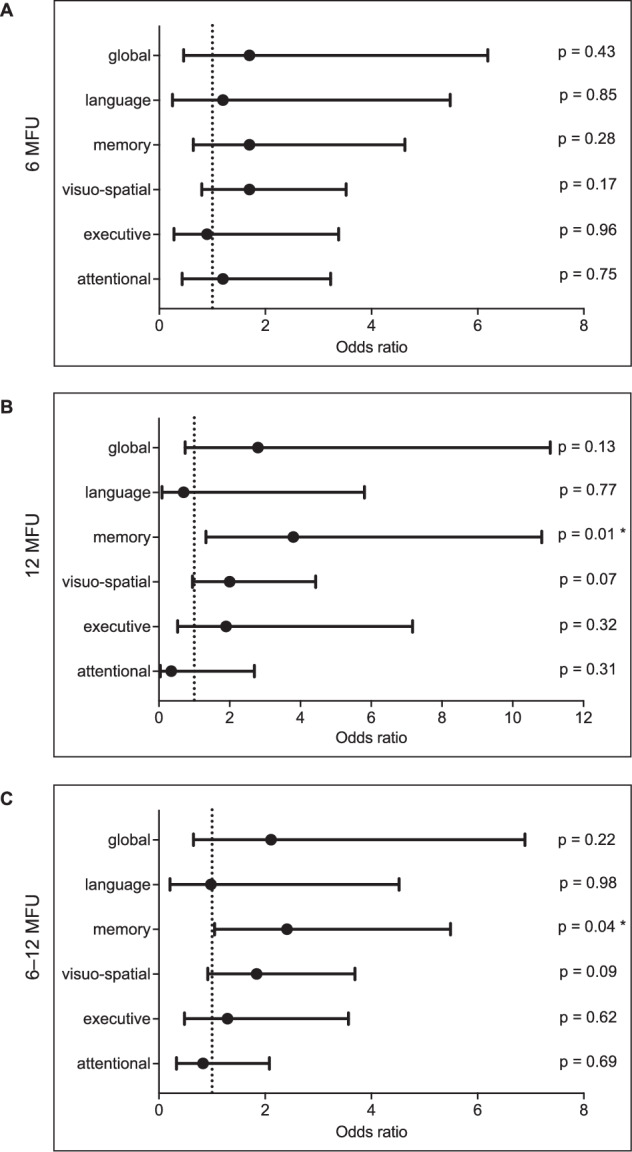
Global and domain-specific cognitive impairment in association to anti-NMDAR1 autoantibody serostatus. Forest plots representing odds ratios (dots) and corresponding 95% confidential intervals (lines) assessing the association of anti-NMDAR1 autoantibody seropositivity and global and domain-specific binary outcomes in propensity score-adjusted logistic regression models at 6-months follow-up (**A**), at 12-months follow-up (**B**), and in logistic GEE analysis from 6- to 12-months follow-up (**C**). Propensity scores were calculated from logistic regression models including age (continuous), sex (dichotomous), education (continuous), ever smoking (dichotomous), habitual alcohol consumption (dichotomous), severe disease (dichotomous), previous stroke or transitory ischemic attack (dichotomous), cardiovascular diseases (dichotomous), and other organic brain diseases (dichotomous), with NMDAR1-abs serostatus as dependent variable. MFU months follow-up. * Indicates statistical significance with the p-value threshold set at <0.05.

With these striking descriptive differences in cognitive test performance, we next analyzed the effect of NMDAR1-abs serostatus on cognitive outcome in stepwise adjusted multiple regression models. Here, neither an effect of NMDAR1-abs serostatus on global cognitive performance, as estimated by linear regression, nor on global cognitive impairment, as estimated by logistic regression models was evident at either timepoint (6-months FU performance: β_Model3_ = −0.05; 95%CI = −0.03 to 0.12, *p* = 0.36 and 6-months FU impairment: OR_Model3_ = 1.7; 95%CI = 0.46 to 6.19, *p* = 0.39; 12-months FU performance: β_Model3_ = −0.04; 95%CI = −0.34 to 0.14, *p* = 0.40 and 12-months FU impairment: OR_Model3_ = 2.8; 95%CI = 0.74 to 11.06, *p* = 0.13). For detailed data, please refer to Fig. 3A, B and Suppl. Tables 3−6.

In domain-specific analyses, at 6-months FU, seropositive patients performed worse in the memory domain compared to seronegative patients (β_Model1_ = −0.10; 95%CI = −0.55 to −0.03, *p* = 0.03; β_Model2_ = −0.09; 95%CI = −0.53 to −0.01, *p* = 0.04; β_Model3_ = −0.09; 95%CI = −0.49 to 0.04, *p* = 0.09) (Suppl. Table 3). However, we observed no distinct effect on memory impairment (OR_Model3_ = 1.7; 95%CI = 0.64 to 4.63, *p* = 0.28) (Fig. 3A and Suppl. Table 5) as determined by our statistical models. NMDAR1-abs serostatus was not associated with any other domain-specific cognitive outcome at 6-months FU, such as language (β_Model3_ = 0.03; 95%CI = −0.18 to 0.36, p = 0.52; Fig. 3A and Suppl. Table 3-4). 12 months after stroke, seropositive patients performed lower in the memory domain (β_Model1_ = −0.10; 95%CI = −0.55 to −0.02, *p* = 0.03, β_Model2_ = −0.10; 95%CI = −0.54 to −0.01, *p* = 0.04, β_Model3_ = −0.11; 95% CI = −0.57 to −0.3, *p* = 0.03), and were at increased risk for memory impairment across all models (OR_Model1_ = 4.8; 95% CI = 1.73 to 13.12, *p* = 0.01, OR_Model2_ = 3.7; 95%CI = 1.28 to 10.41, *p* = 0.02, OR_Model3_ = 3.8; 95%CI = 1.33 to 10.82, *p* = 0.01; Fig. 3B and Suppl. Tables 4 + 6). This association was exclusively evident for the memory domain (Fig. 3B and Suppl. Table 4 + 6). In addition, over 6- to 12-months FU together, the effect of NMDAR1-abs on memory impairment remained consistent (OR_Model3_ = 2.4; 95%CI = 1.05 to 5.49, *p* = 0.04; Fig. 3C and Suppl. Table 7). Again, this link was not observed in any other cognitive domain (e.g., language OR_Model3_ = 0.98; 95%CI = 0.21 to 4.52, *p* = 0.98; Fig. 3C and Suppl. Table 7). Although the point estimate of the GEE analysis suggested that NMDAR1-abs seropositive patients have an increased risk for global cognitive impairment, this direction was not conclusively confirmed by the confidence interval, and the data were too imprecise to draw conclusions (OR_Model3_ = 2.11; 95%CI = 0.65 to 6.88, *p* = 0.22) (Fig. 3C). The complete data are presented in Suppl. Table 7.

### Post-hoc exploration of the relationship between other neuropsychiatric outcomes and baseline hippocampal volume with memory performance during follow-up

**Fig. 4:**
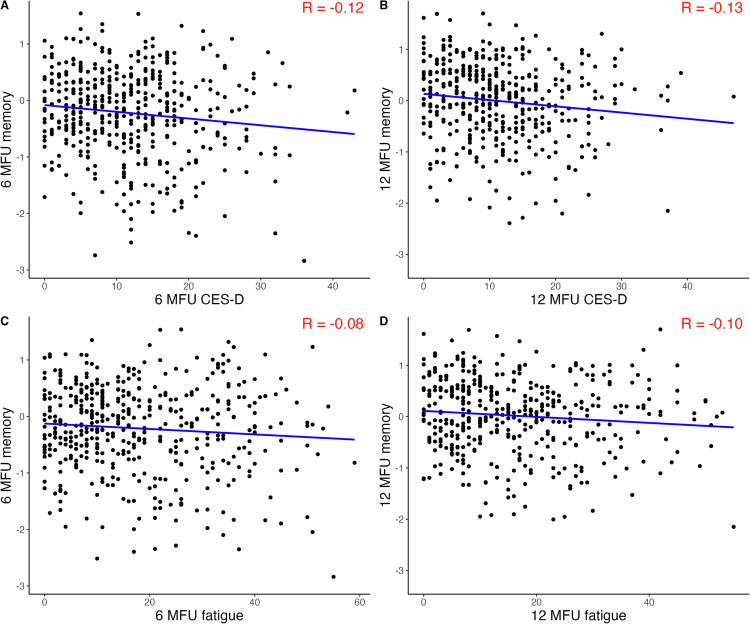
Scatter plots and correlation coefficients for depression and fatigue with memory function at both follow-up timepoints. **A** + **B** x-axis: Center for Epidemiological Studies Depression (CES-D), y-axis: z-scores for memory function at 6- and 12-months, respectively. R: correlation coefficient. **C** + **D** x-axis: Fatigue Assessment Questionnaire (FAQ), y-axis: z-scores for memory function at 6- and 12-months respectively. R correlation coefficient, MFU months follow-up.

**Fig. 5:**
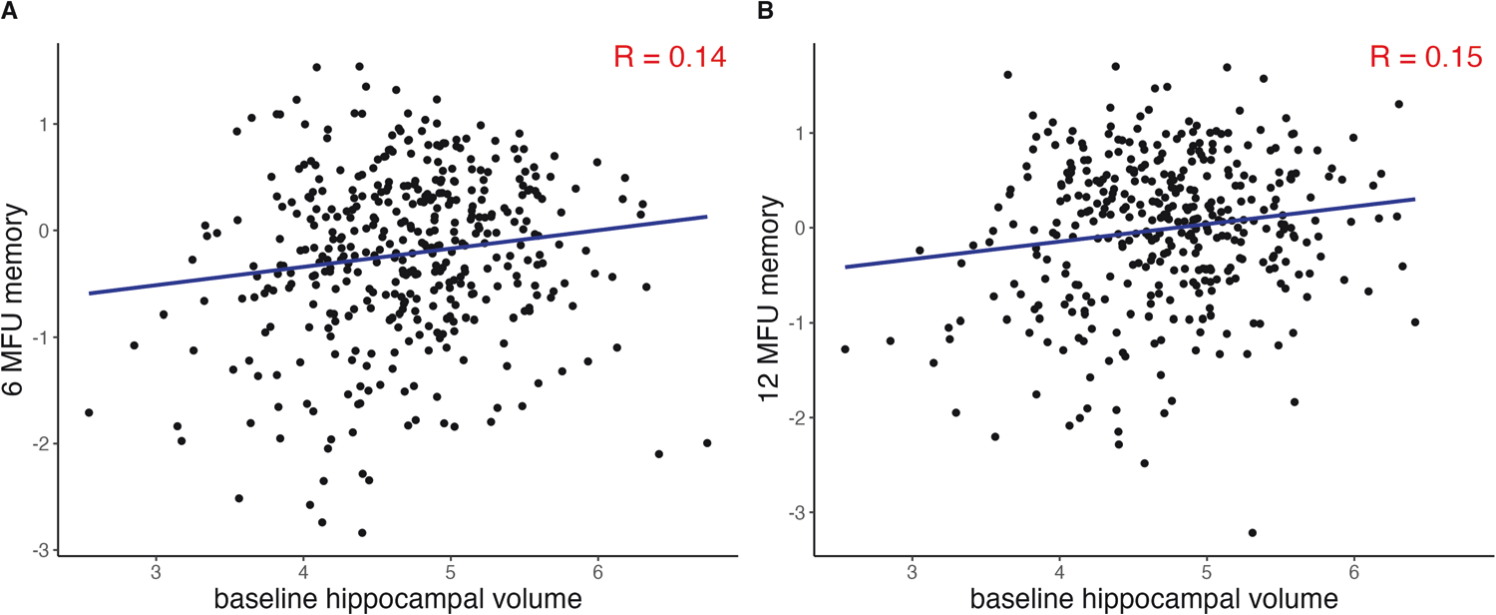
Scatter plots and correlation coefficients for baseline hippocampal volumes with memory function at both follow-up timepoints: X-axis. Baseline hippocampal volume, y-axis: z-scores for memory function at (**A**) 6- and (**B**) 12-months, R correlation coefficient, MFU months follow-up.

Mean CES-D and mean FAQ appeared to be similar in seropositive and seronegative patients during follow-up (Suppl. Table 8). We visually explored the linear relationship of depressive symptoms (CES-D) and fatigue symptoms (FAQ) with z-scores of the memory tests and calculated correlation coefficients, which rendered low correlation of these two other neuropsychiatric outcomes at both timepoints (Fig. 4). Additionally, hippocampal volumes at baseline did not show a strong correlation with memory z-scores at both follow-up timepoints (Fig. 5).

## Discussion

In this analysis of the DEMDAS stroke cohort, serum prevalence of NMDAR1-abs, mainly of the IgM and IgA isotype, was 10.2%. Seropositivity for NMDAR1-abs was associated with poorer memory performance compared to seronegative patients and with memory impairment at 12 months post-stroke, as well as from 6 to 12 months combined. In contrast, seropositivity was not associated with global cognitive function or impairment, nor with any of the other cognitive subdomains.

While the proportion of NMDAR1-abs serum prevalence varied between 13 and 23% in other stroke cohorts [12, 38], the prevalence of NMDAR1-abs in our study (10%) was somewhat lower. Similar to another study, serum prevalence was higher in older and in male patients [39]. The high frequency of NMDAR1-abs in healthy individuals and patients with different diseases, calls into question a pathological significance of seropositivity per se. Following ischemic stroke the blood brain barrier is at least focally disrupted, potentially allowing antibodies to access the brain [40]. In agreement with others, we therefore hypothesize that damage to the blood brain barrier may be an additional necessary factor for NMDAR1-abs to exert pathological effects [41–43].

Our data extend recent findings linking serum prevalence of NMDAR1-abs and neuropsychiatric outcome following stroke [11, 12]. In contrast to a recent work [12], we found no clear association of seropositivity with global cognitive function after stroke in this study. This could be explained by a different duration of follow-up (3 years vs. 1 year), as in our study, effects became only clearly apparent after 12 months (not yet after 6 months). Our current and previous data therefore suggest that effects of serum NMDAR1-IgAs and -IgMs may manifest only over time after stroke. A possible explanation could be an antibody-mediated downregulation of NMDA-receptors with subsequent effects on synaptic plasticity and thereby on long-term regenerative processes after stroke [44–46].

An association between NMDAR1-abs and decreased cognitive abilities has been established in several other cohorts and various disorders [8, 14–16]. It is plausible though that previously observed associations of NMDAR1-abs with global cognitive performance are mainly attributable to dysfunction in the memory domain, as supported by findings regarding immediate memory, from one respective study [12].

The link between NMDAR1-abs seropositivity and reduced memory function is particularly intriguing and biologically plausible given the high density of NMDA-receptors in the hippocampus. In fact, we found slightly smaller hippocampal volumes in seropositive patients already at baseline. However, the effect of seropositivity on the memory impairment was only evident after 12 months, and correlations of memory z-scores at follow-up timepoints and hippocampal volumes at baseline were low. Baseline hippocampal volumes therefore do not appear as the main factor for memory function in the long term after stroke. However, whether seropositivity impacts hippocampal volumes over time upon blood brain barrier impairment due to stroke remains to be determined. While some studies question whether NMDA-receptors are internalized after binding of IgA/M NMDAR1-abs [47, 48], evidence shows at least IgA binding to hippocampal NMDA receptors, however, with lower affinity than IgG, possibly leading to local ‘latent’ autoimmunity [14, 41, 49]. Effects on memory performance may be primarily attributed to NMDAR1-abs IgA antibodies, as observed differences in our cohort were most pronounced in patients with IgA isotype antibodies. However, the sample size was too small to further investigate the effects by isotype stratification.

NMDAR1-abs seropositive patients in our study were more likely to have ischemic cerebrovascular events before the index stroke compared with seronegative patients (19% vs. 12%). This may suggest that seropositive patients have a higher baseline risk of cerebrovascular events [7], or that NMDAR1-abs formation is induced by cerebrovascular events, although other studies have challenged this notion [7, 11, 43].

Cardiovascular risk factors as potential confounders did not lead to major changes in point estimates in our memory domain analyses. In line with previous work [50], this suggests that vascular risk factors do not particularly influence memory function. In contrast, intravenous thrombolytic therapy could modify the effect of NMDAR1-abs on cognitive outcomes, as tissue-type plasminogen activator (tPA) has been shown to affect blood brain barrier integrity and neuroinflammation as well as neuronal survival by altering NMDAR signaling in endothelial and neuronal cells [51]. Since our analysis revealed too large CIs after excluding patients treated with rtPA (data not shown), future studies with larger cohorts should investigate the role of rtPA in this context.

Interestingly, neuropsychiatric outcomes (i.e., depression, fatigue) did not appear to be substantially different in NMDAR1-abs seropositive compared to seronegative patients at either follow-up timepoint, which is in contrast to previous study results [12, 52]. Since these outcomes were generally only weakly correlated with memory function in our study, we infer that memory function is not majorly impacted by these conditions, highlighting a specific and independent mechanisms despite additional neuropsychiatric findings.

In a previous analysis of the DEMDAS cohort, we found a robust connection between small vessel disease burden in baseline MRI and cognitive impairment at follow-up timepoints at global and subdomain levels, with exception of the memory domain [23]. This again suggests distinct underlying mechanisms leading to memory impairment vs. impairment in other cognitive domains after stroke. Taken together, our data might reveal a novel subtype of post-stroke cognitive impairment characterized by memory dysfunction, however exact mechanisms of post-stroke inflammation and potential autoimmunity remain unclear [53].

### Strengths and limitations

A clear strength of the study is the detailed neuropsychological testing with the CERAD battery and the size of the prospectively analyzed cohort. However, the relatively small number of seropositive patients (*n* = 58) limits the precision of our estimates as indicated by large CIs. Another major limitation is the short follow-up time and the limited access to imaging data as no MRI was acquired after 12 months. Future investigation of domain-specific cognitive outcomes and concurrent data on hippocampal volumes over time might elucidate imaging correlates of memory impairment in NMDAR1-abs seropositivity after stroke. The frequency of missing data from patients at 6 and 12 months was 16.8% and 22.8%, respectively, which is not unusual in observational studies. Although baseline characteristics were not different between patients who dropped out of the study and those who remained in the study, lost-to-follow-up may be differential. In this line, follow-up rates were higher in seronegative participants than in seropositive patients.

Our data add to a growing body of evidence derived from clinical observations pointing towards a functional relevance of NMDAR1-abs for cognitive outcomes after stroke, particularly memory function. The underlying distinct pathophysiological mechanisms need to be studied in the future using experimental stroke models and large patient cohorts.

## Supplementary information

Serum anti-NMDA receptor antibodies are linked to memory impairment 12 months after stroke – Supplementary Material

## Data Availability

All primary data and analyses scripts are available from the responsible principal investigator (matthias.endres@charite.de) upon reasonable request.

## References

[1] Collaborators GBDS. Global, regional, and national burden of stroke, 1990-2016: a systematic analysis for the Global Burden of Disease Study 2016. Lancet Neurol. 2019;18:439–58. 30871944 10.1016/S1474-4422(19)30034-1 PMC6494974

[2] Pendlebury ST, Rothwell PM. Prevalence, incidence, and factors associated with pre-stroke and post-stroke dementia: a systematic review and meta-analysis. Lancet Neurol. 2009;8:1006–18. 19782001 10.1016/S1474-4422(09)70236-4

[3] Pendlebury ST, Rothwell PM, Oxford VS. Incidence and prevalence of dementia associated with transient ischaemic attack and stroke: analysis of the population-based Oxford Vascular Study. Lancet Neurol. 2019;18:248–58. 30784556 10.1016/S1474-4422(18)30442-3 PMC6390174

[4] Dichgans M, Zietemann V. Prevention of vascular cognitive impairment. Stroke. 2012;43:3137–46. 22935401 10.1161/STROKEAHA.112.651778

[5] Dahm L, Ott C, Steiner J, Stepniak B, Teegen B, Saschenbrecker S, et al. Seroprevalence of autoantibodies against brain antigens in health and disease. Ann Neurol. 2014;76:82–94. 10.1002/ana.24189 24853231

[6] Steiner J, Teegen B, Schiltz K, Bernstein HG, Stoecker W, Bogerts B. Prevalence of N-methyl-D-aspartate receptor autoantibodies in the peripheral blood: healthy control samples revisited. JAMA Psychiatry. 2014;71:838–9. 24871043 10.1001/jamapsychiatry.2014.469

[7] Sperber PS, Siegerink B, Huo S, Rohmann JL, Piper SK, Pruss H, et al. Serum anti-NMDA (N-methyl-D-aspartate)-receptor antibodies and long-term clinical outcome after stroke (PROSCIS-B). Stroke. 2019;50:3213–9. 31526121 10.1161/STROKEAHA.119.026100

[8] Doss S, Wandinger KP, Hyman BT, Panzer JA, Synofzik M, Dickerson B, et al. High prevalence of NMDA receptor IgA/IgM antibodies in different dementia types. Ann Clin Transl Neurol. 2014;1:822–32. 25493273 10.1002/acn3.120 PMC4241809

[9] During MJ, Symes CW, Lawlor PA, Lin J, Dunning J, Fitzsimons H, et al. An oral vaccine against NMDARI with efficacy in experimental stroke and epilepsy. Science. 2000;287:1453–60. 10.1126/science.287.5457.1453 10688787

[10] Zerche M, Weissenborn K, Ott C, Dere E, Asif AR, Worthmann H, et al. Preexisting serum autoantibodies against the NMDAR subunit NR1 modulate evolution of lesion size in acute ischemic stroke. Stroke. 2015;46:1180–6. 25765725 10.1161/STROKEAHA.114.008323

[11] Sperber PS, Gebert P, Broersen LHA, Huo S, Piper SK, Teegen B, et al. Serum anti-NMDA-receptor antibodies and cognitive function after ischemic stroke (PROSCIS-B). J Neurol. 2022;269:5521–30. 35718820 10.1007/s00415-022-11203-x PMC9468072

[12] Deutsch NR, Worthmann H, Steixner-Kumar AA, Schuppner R, Grosse GM, Pan H, et al. Autoantibodies against the NMDAR subunit NR1 are associated with neuropsychiatric outcome after ischemic stroke. Brain Behav Immun. 2021;96:73–9. 34010714 10.1016/j.bbi.2021.05.011

[13] Banwell B, Bennett JL, Marignier R, Kim HJ, Brilot F, Flanagan EP, et al. Diagnosis of myelin oligodendrocyte glycoprotein antibody-associated disease: international MOGAD Panel proposed criteria. Lancet Neurol. 2023;22:268–82. 36706773 10.1016/S1474-4422(22)00431-8

[14] Pruss H, Holtje M, Maier N, Gomez A, Buchert R, Harms L, et al. IgA NMDA receptor antibodies are markers of synaptic immunity in slow cognitive impairment. Neurology. 2012;78:1743–53. 22539565 10.1212/WNL.0b013e318258300d PMC3359581

[15] Bartels F, Stronisch T, Farmer K, Rentzsch K, Kiecker F, Finke C. Neuronal autoantibodies associated with cognitive impairment in melanoma patients. Ann Oncol. 2019;30:823–9. 30840061 10.1093/annonc/mdz083 PMC6551450

[16] Finke C, Bartels F, Lutt A, Pruss H, Harms L. High prevalence of neuronal surface autoantibodies associated with cognitive deficits in cancer patients. J Neurol. 2017;264:1968–77. 28785798 10.1007/s00415-017-8582-0

[17] Ehrenreich H, Wilke J, Steixner-Kumar AA. Spontaneous serum autoantibody fluctuations: To be or not to be. Mol Psychiatry. 2021;26:1723–5. 32968237 10.1038/s41380-020-00883-4 PMC8440174

[18] Hammer C, Stepniak B, Schneider A, Papiol S, Tantra M, Begemann M, et al. Neuropsychiatric disease relevance of circulating anti-NMDA receptor autoantibodies depends on blood-brain barrier integrity. Mol Psychiatry. 2014;19:1143–9. 23999527 10.1038/mp.2013.110

[19] Wollenweber FA, Zietemann V, Rominger A, Opherk C, Bayer-Karpinska A, Gschwendtner A, et al. The determinants of dementia after stroke (DEDEMAS) study: protocol and pilot data. Int J Stroke. 2014;9:387–92. 23834337 10.1111/ijs.12092

[20] Jorm AF. A short form of the Informant Questionnaire on Cognitive Decline in the Elderly (IQCODE): development and cross-validation. Psychol Med. 1994;24:145–53. 8208879 10.1017/s003329170002691x

[21] Ramberger M, Peschl P, Schanda K, Irschick R, Hoftberger R, Deisenhammer F, et al. Comparison of diagnostic accuracy of microscopy and flow cytometry in evaluating N-methyl-D-aspartate receptor antibodies in serum using a live cell-based assay. PLoS One. 2015;10:e0122037. 25815887 10.1371/journal.pone.0122037 PMC4376531

[22] Berres M, Monsch AU, Bernasconi F, Thalmann B, Stähelin HB. Normal ranges of neuropsychological tests for the diagnosis of Alzheimer’s disease. Stud Health Technol Inform. 2000;77:195–9. 11187541

[23] Georgakis MK, Fang R, During M, Wollenweber FA, Bode FJ, Stosser S, et al. Cerebral small vessel disease burden and cognitive and functional outcomes after stroke: A multicenter prospective cohort study. Alzheimers Dement. 2023;19:1152–63. 35876563 10.1002/alz.12744

[24] Folstein MF, Folstein SE, Mchugh PR. “Mini-mental state”. A practical method for grading the cognitive state of patients for the clinician. J Psychiatr Res. 1975;12:189–98. 10.1016/0022-3956(75)90026-6 1202204

[25] Somerville J, Tremont G, Stern RA. The Boston Qualitative Scoring System as a measure of executive functioning in Rey-Osterrieth Complex Figure performance. J Clin Exp Neuropsychol. 2000;22:613–21. 11094396 10.1076/1380-3395(200010)22:5;1-9;FT613

[26] Bäumler G. Farb-Wort-Interferenztest (FWIT) nach J.R. Stroop. Germany: Hogrefe Göttingen; 1985.

[27] Young JC, Sawyer RJ, Roper BL, Baughman BC. Expansion and re-examination of Digit Span effort indices on the WAIS-IV. Clin Neuropsychol. 2012;26:147–59. 22268525 10.1080/13854046.2011.647083

[28] Knopman DS, Beiser A, Machulda MM, Fields J, Roberts RO, Pankratz VS, et al. Spectrum of cognition short of dementia Framingham heart study and Mayo Clinic study of aging. Neurology. 2015;85:1712–21. 10.1212/WNL.0000000000002100 PMC4653114 26453643

[29] Nasreddine ZS, Phillips NA, Bédirian V, Charbonneau S, Whitehead V, Collin I, et al. The Montreal Cognitive Assessment, MoCA: a brief screening tool for mild cognitive impairment. J Am Geriatr Soc. 2005;53:695–9. 10.1111/j.1532-5415.2005.53221.x 15817019

[30] Zietemann V, Georgakis MK, Dondaine T, Muller C, Mendyk AM, Kopczak A, et al. Early MoCA predicts long-term cognitive and functional outcome and mortality after stroke. Neurology. 2018;91:e1838–e50. 30333158 10.1212/WNL.0000000000006506

[31] Burton L, Tyson SF. Screening for cognitive impairment after stroke: a systematic review of psychometric properties and clinical utility. J Rehabil Med. 2015;47:193–203. 25590458 10.2340/16501977-1930

[32] Frick J, Gebert P, Grittner U, Letsch A, Schindel D, Schenk L. Identifying and handling unbalanced baseline characteristics in a non-randomized, controlled, multicenter social care nurse intervention study for patients in advanced stages of cancer. BMC Cancer. 2022;22:560. 35585571 10.1186/s12885-022-09646-6 PMC9118792

[33] Jahn R, Baumgartner JS, van den Nest M, Friedrich F, Alexandrowicz RW, Wancata J. [Criterion validity of the German version of the CES-D in the general population]. Psychiatr Prax. 2018;45:434–42. 29665610 10.1055/a-0584-9803

[34] Parikh RM, Eden DT, Price TR, Robinson RG. The sensitivity and specificity of the Center for Epidemiologic Studies Depression Scale in screening for post-stroke depression. Int J Psychiatry Med. 1988;18:169–81. 3170080 10.2190/bh75-euya-4fm1-j7qa

[35] Glaus A, Crow R, Hammond S. A qualitative study to explore the concept of fatigue/tiredness in cancer patients and in healthy individuals. Support Care Cancer. 1996;4:82–96. 8673356 10.1007/BF01845757

[36] Mead G, Lynch J, Greig C, Young A, Lewis S, Sharpe M. Evaluation of fatigue scales in stroke patients. Stroke. 2007;38:2090–5. 17525397 10.1161/STROKEAHA.106.478941

[37] von Elm E, Altman DG, Egger M, Pocock SJ, Gotzsche PC, Vandenbroucke JP, et al. The Strengthening the Reporting of Observational Studies in Epidemiology (STROBE) statement: guidelines for reporting observational studies. Lancet. 2007;370:1453–7. 18064739 10.1016/S0140-6736(07)61602-X

[38] Royl G, Fokou TJ, Chunder R, Isa R, Munte TF, Wandinger KP, et al. Antibodies against neural antigens in patients with acute stroke: joint results of three independent cohort studies. J Neurol. 2019;266:2772–9. 31359201 10.1007/s00415-019-09470-2

[39] Daguano Gastaldi V, Bh Wilke J, Weidinger CA, Walter C, Barnkothe N, Teegen B, et al. Factors predisposing to humoral autoimmunity against brain-antigens in health and disease: Analysis of 49 autoantibodies in over 7000 subjects. Brain Behav Immun. 2023;108:135–47. 36323361 10.1016/j.bbi.2022.10.016

[40] Pruss H, Iggena D, Baldinger T, Prinz V, Meisel A, Endres M, et al. Evidence of intrathecal immunoglobulin synthesis in stroke: a cohort study. Arch Neurol. 2012;69:714–7. 22371852 10.1001/archneurol.2011.3252

[41] Ehrenreich H. Autoantibodies against N-methyl-d-aspartate receptor 1 in health and disease. Curr Opin Neurol. 2018;31:306–12. 29474316 10.1097/WCO.0000000000000546 PMC5959203

[42] Castillo-Gomez E, Oliveira B, Tapken D, Bertrand S, Klein-Schmidt C, Pan H, et al. All naturally occurring autoantibodies against the NMDA receptor subunit NR1 have pathogenic potential irrespective of epitope and immunoglobulin class. Mol Psychiatry. 2017;22:1776–84. 27502473 10.1038/mp.2016.125

[43] Pan H, Steixner-Kumar AA, Seelbach A, Deutsch N, Ronnenberg A, Tapken D, et al. Multiple inducers and novel roles of autoantibodies against the obligatory NMDAR subunit NR1: a translational study from chronic life stress to brain injury. Mol Psychiatry. 2021;26:2471–82. 32089545 10.1038/s41380-020-0672-1 PMC8440197

[44] Wenke NK, Kreye J, Andrzejak E, van Casteren A, Leubner J, Murgueitio MS, et al. N-methyl-D-aspartate receptor dysfunction by unmutated human antibodies against the NR1 subunit. Ann Neurol. 2019;85:771–6. 30843274 10.1002/ana.25460 PMC6593665

[45] Jaenisch N, Liebmann L, Guenther M, Hubner CA, Frahm C, Witte OW. Reduced tonic inhibition after stroke promotes motor performance and epileptic seizures. Sci Rep. 2016;6:26173. 27188341 10.1038/srep26173 PMC4870642

[46] Hardingham GE, Bading H. Synaptic versus extrasynaptic NMDA receptor signalling: implications for neurodegenerative disorders. Nat Rev Neurosci. 2010;11:682–96. 20842175 10.1038/nrn2911 PMC2948541

[47] Hara M, Martinez-Hernandez E, Arino H, Armangue T, Spatola M, Petit-Pedrol M, et al. Clinical and pathogenic significance of IgG, IgA, and IgM antibodies against the NMDA receptor. Neurology. 2018;90:e1386–e94. 29549218 10.1212/WNL.0000000000005329 PMC5902781

[48] Dalmau J. Letter by Dalmau Regarding Article, “Serum anti-NMDA (N-methyl-D-aspartate)-receptor antibodies and long-term clinical outcome after stroke (PROSCIS-B)”. Stroke. 2020;51:e28. 31928149 10.1161/STROKEAHA.119.027885

[49] Prüss H. Autoantibodies in neurological disease. Nat Rev Immunol. 2021;21:798–813. 33976421 10.1038/s41577-021-00543-w PMC8111372

[50] Sachdev PS, Brodaty H, Valenzuela MJ, Lorentz L, Looi JC, Wen W, et al. The neuropsychological profile of vascular cognitive impairment in stroke and TIA patients. Neurology. 2004;62:912–9. 15037692 10.1212/01.wnl.0000115108.65264.4b

[51] Lebrun F, Levard D, Lemarchand E, Yetim M, Furon J, Potzeha F, et al. Improving stroke outcomes in hyperglycemic mice by modulating tPA/NMDAR signaling to reduce inflammation and hemorrhages. Blood Adv. 2024;8:1330–44. 38190586 10.1182/bloodadvances.2023011744 PMC10943589

[52] Sperber PS, Gebert P, Broersen LHA, Kufner A, Huo S, Piper SK, et al. Depressive symptoms and anti-N-methyl-D-aspartate-receptor GluN1 antibody seropositivity in the PROSpective cohort with incident stroke. Brain Behav Immun Health. 2023;34:100705. 38033615 10.1016/j.bbih.2023.100705 PMC10684375

[53] Endres M, Moro MA, Nolte CH, Dames C, Buckwalter MS, Meisel A. Immune Pathways in Etiology, Acute Phase, and Chronic Sequelae of Ischemic Stroke. Circ Res. 2022;130:1167–86. 10.1161/CIRCRESAHA.121.319994 35420915

