# Supplementary material for: Serum anti-NMDA receptor antibodies are linked to memory impairment 12 months after stroke
Source: Mol Psychiatry. 2024 Oct 30;30(4):1359–68. doi: 10.1038/s41380-024-02744-w (PMC11919755; doi:10.1038/s41380-024-02744-w)
Supplement: Supplementary file 1 — Serum anti-NMDA receptor antibodies are linked to memory impairment 12 months after stroke – Supplementary Material [file 41380_2024_2744_MOESM1_ESM.docx]

**Serum anti-NMDA receptor antibodies are linked to memory impairment 12 months after stroke**

Friederike A. Arlt and Pia S. Sperber et al.

Supplementary Table 1

| Department and Location of participating Centers |
| --- |
| Department of Neurology, University Hospital, LMU, Munich Germany |
| Department of Neurology, Klinikum rechts der Isar, School of Medicine, Technical University of Munich, Munich Germany |
| Division of Vascular Neurology, Department of Neurology, University Hospital Bonn, Bonn Germany |
| University Medical Center, the Department of Neurology, Göttingen, Göttingen Germany |
| Department of Neurology and Institute of Cognitive Neurology and Dementia Research, Otto von Guericke University Magdeburg, Magdeburg Germany |
| Center for Stroke Research Berlin, Charité – Universitätsmedizin Berlin, Berlin Germany |
| Department of Neurology, Charité – Universitätsmedizin Berlin, Berlin Germany |

**Supplementary Table 1: Participating Centers of the DEMDAS study**

Supplementary Table 2

|  | 6-months FU | | | 12-months FU | | |
| --- | --- | --- | --- | --- | --- | --- |
| cognitive domain with impairment (yes/no) | DEMDAS  n (%) | seronegative  n (%) | seropositive  n (%) | DEMDAS  n (%) | seronegative  n (%) | seropositive  n (%) |
| global | 21/451 (4.7) | 18/409 (4.4) | 3/37 (8.1) | 14/423 (3.3) | 11/382 (2.9) | 3/36 (8.3) |
| language | 19/470 (4.0) | 17/425 (4.0) | 2/40 (5.0) | 15/432 (3.5) | 14/390 (3.6) | 1/37 (2.7) |
| memory | 38/470 (8.1) | 32/425 (7.5) | 6/40 (15.0) | 21/439 (4.8) | 15/396 (3.8) | 6/38 (15.8) |
| visuo-spatial | 94/471 (20) | 81/426 (19.0) | 13/40 (32.5) | 72/439 (16.4) | 61/396 (15.4) | 11/38 (28.9) |
| executive | 36/453 (7.9) | 33/411 (8.0) | 3/37 (8.1) | 22/423 (5.2) | 19/382 (5.0) | 3/36 (8.3) |
| attentional | 47/463(10.2) | 42/419 (10.0) | 5/39 (12.8) | 24/430 (5.6) | 23/388 (5.9) | 1/37 (2.7) |

**Supplementary Table 2: Cognitive impairment at 6- and 12-months follow-up stratified upon anti-NMDA-receptor GluN1 autoantibody serostatus**. Impairment defined as z-score <-1.5. FU: follow-up.

Supplementary Figure 1


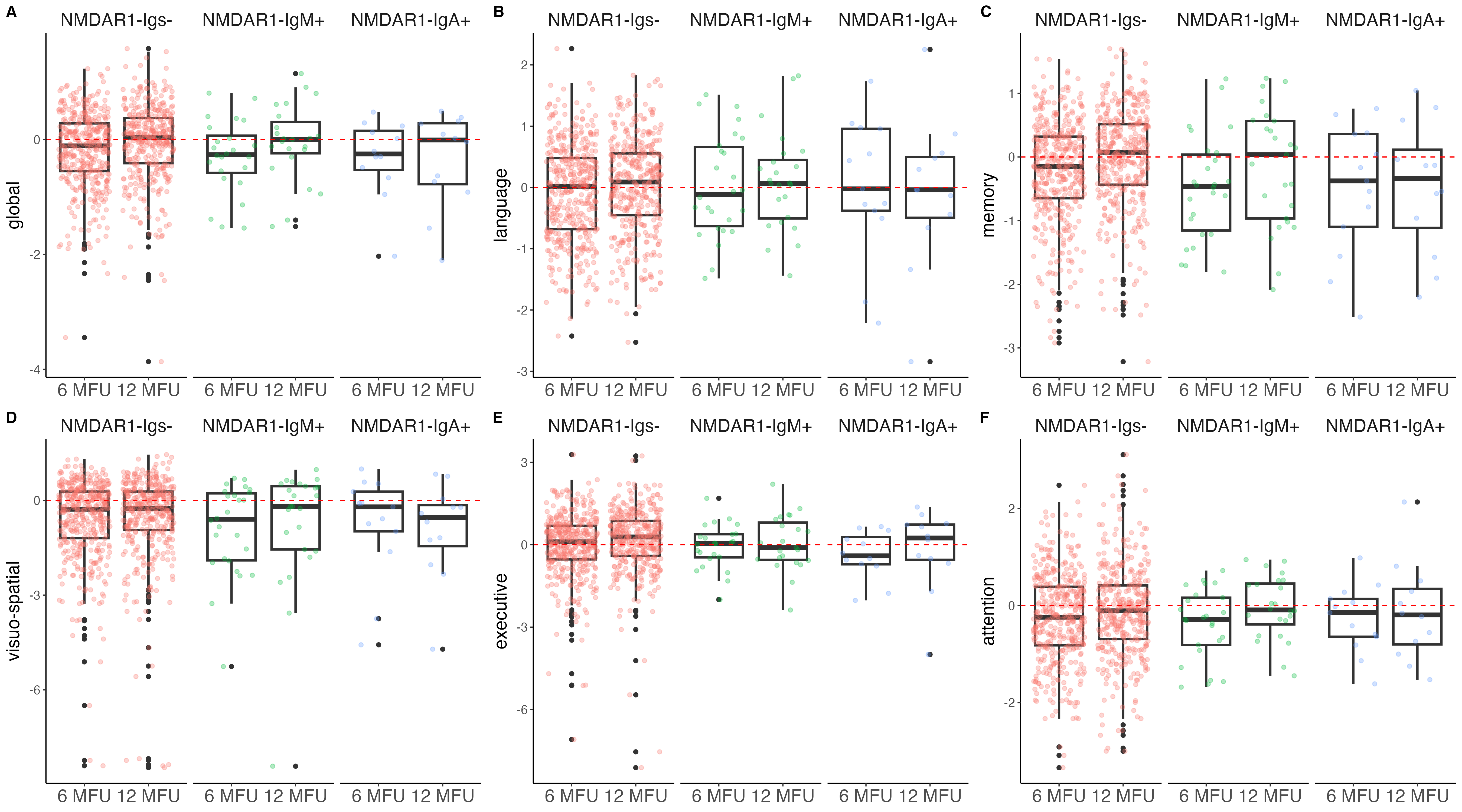


**Supplementary Figure 1: Global and subdomain scoring from the Consortium to Establish a Registry for Alzheimer’s Disease Plus’– cognitive test battery (CERAD-Plus) stratified by different anti-NMDA-receptor GluN1 antibody (NMDAR1-abs) immunoglobulin (Ig-)isotypes:**

Red dots represent single participants’ z-scores of NMDAR1-abs seronegative patients while green (IgM) and purple (IgA) dots display single participants’ z-scores of NMDAR1 autoantibody seropositive patients with a boxplot overlay and emphasized zero-line (red dashed line). Age, sex, and education standardized z-scores were calculated from a reference normative population. MFU: months follow-up

Supplementary Table 3

|  | **6-months FU** | | | | | | | | |
| --- | --- | --- | --- | --- | --- | --- | --- | --- | --- |
|  | Model 1 | | | Model 2 | | | Model 3 | | |
|  | **β** | **95% CI** | **p-value** | **β** | **95% CI** | **p-value** | **β** | **95% CI** | **p-value** |
| global | **-0.05** | -0.35 – 0.09 | 0.25 | **-0.05** | -0.35 – 0.09 | 0.27 | **-0.05** | -0.33 – 0.12 | 0.36 |
| language | **0.03** | -0.17 – 0.35 | 0.50 | **0.02** | -0.19 – 0.33 | 0.63 | **0.03** | -0.18 – 0.36 | 0.52 |
| memory | **-0.10** | -0.55 – ‑0.03 | 0.03 | **-0.09** | -0.53 – ‑0.01 | 0.04 | **-0.09** | -0.49 – 0.04 | 0.09 |
| visuo-spatial | **-0.07** | -0.73 – 0.09 | 0.13 | **-0.05** | -0.64 – 0.17 | 0.25 | **-0.05** | -0.63 – 0.22 | 0.33 |
| executive | **-0.03** | -0.52 – 0.24 | 0.48 | **-0.03** | -0.51 – 0.27 | 0.54 | **-0.03** | -0.52 – 0.24 | 0.48 |
| attentional | **-0.02** | -0.37 – 0.23 | 0.67 | **-0.01** | -0.30 – 0.27 | 0.86 | **-0.01** | -0.27 – 0.34 | 0.82 |

**Supplementary Table 3:** **Effect estimates of anti-NMDA-receptor GluN1 antibody serostatus on cognitive performance from stepwise adjusted linear regression models at 6-months following stroke.** Model 1: crude, no adjustment; Model 2: adjusted for age (continuous), sex (dichotomous), and educational years (continuous), Model 3: propensity score adjusted models accounting for confounding factors. Propensity scores were calculated from logistic regression models including age (continuous), sex (dichotomous), education (continuous), ever smoking (dichotomous), habitual alcohol consumption (dichotomous), severe disease (dichotomous), previous stroke or transitory ischemic attack (dichotomous), cardiovascular diseases (dichotomous), and other organic brain diseases (dichotomous), with NMDAR1-abs serostatus as dependent variable. 95% CI: 95% confidential interval, FU: follow-up.

Supplementary Table 4

|  | **12-months FU** | | | | | | | | |
| --- | --- | --- | --- | --- | --- | --- | --- | --- | --- |
|  | Model 1 | | | Model 2 | | | Model 3 | | |
|  | **β** | **95% CI** | **p-value** | **β** | **95% CI** | **p-value** | **β** | **95% CI** | **p-value** |
| global | **-0.05** | -0.35 – 0.12 | 0.33 | **-0.04** | -0.34 – 0.13 | 0.39 | **-0.04** | -0.34 – 0.14 | 0.40 |
| language | **0.01** | -0.25 – 0.29 | 0.88 | **0.01** | -0.27 – 0.27 | 0.99 | **0.01** | -0.27 – 0.29 | 0.93 |
| memory | **-0.10** | -0.55 – ‑0.02 | 0.03 | **-0.10** | -0.54 – ‑0.01 | 0.04 | **-0.11** | -0.57 – ‑0.03 | 0.03 |
| visuo-spatial | **-0.06** | -0.81 – 0.15 | 0.18 | **-0.05** | -0.73 – 0.22 | 0.29 | **-0.05** | 0.75 – 0.23 | 0.29 |
| executive | **-0.04** | -0.57 – 0.24 | 0.43 | **-0.04** | -0.56 – 0.26 | 0.47 | **-0.04** | -0.56 – 0.25 | 0.45 |
| attentional | **0.03** | -0.27 – 0.39 | 0.59 | **0.05** | -0.15 – 0.47 | 0.31 | **0.06** | -0.11 – 0.52 | 0.21 |

**Supplementary Table 4:** **Effect estimates of anti-NMDA-receptor GluN1 antibody serostatus on cognitive performance from stepwise adjusted linear regression models at 12-months following stroke.** Model 1: crude, no adjustment; Model 2: adjusted for age (continuous), sex (dichotomous), and educational years (continuous), Model 3: propensity score adjusted models accounting for confounding factors. Propensity scores were calculated from logistic regression models including age (continuous), sex (dichotomous), education (continuous), ever smoking (dichotomous), habitual alcohol consumption (dichotomous), severe disease (dichotomous), previous stroke or transitory ischemic attack (dichotomous), cardiovascular diseases (dichotomous), and other organic brain diseases (dichotomous), with NMDAR1-abs serostatus as dependent variable. 95% CI: 95% confidential interval, FU: follow-up.

Supplementary Table 5

|  | 6-months FU | | | | | | | | |
| --- | --- | --- | --- | --- | --- | --- | --- | --- | --- |
|  | Model 1 | | | Model 2 | | | Model 3 | | |
|  | **OR** | **95% CI** | **p-value** | **OR** | **95% CI** | **p-value** | **OR** | **95% CI** | **p-value** |
| global | **1.9** | 0.54 – 6.83 | 0.32 | **1.8** | 0.48 – 6.49 | 0.39 | **1.7** | 0.46 – 6.19 | 0.43 |
| language | **1.3** | 0.29 – 5.68 | 0.76 | **1.4** | 0.29 – 5.64 | 0.68 | **1.2** | 0.25 – 5.48 | 0.85 |
| memory | **2.2** | 0.85 – 5.55 | 0.11 | **2.0** | 0.77 – 5.45 | 0.15 | **1.7** | 0.64 – 4.63 | 0.28 |
| visuo-spatial | **2.1** | 1.01 – 4.15 | 0.05 | **1.9** | 0.90 – 4.17 | 0.09 | **1.7** | 0.80 – 3.52 | 0.17 |
| executive | **1.0** | 0.29 – 3.47 | 0.99 | **0.9** | 0.28 – 3.35 | 0.96 | **0.9** | 0.28 – 3.38 | 0.96 |
| attentional | **1.3** | 0.49 – 3.59 | 0.58 | **1.4** | 0.51 – 3.84 | 0.52 | **1.2** | 0.43 – 3.23 | 0.75 |

**Supplementary Table 5:** **Effect estimates of anti-NMDA-receptor GluN1 antibody serostatus on cognitive impairment from stepwise adjusted logistic regression models at 6-months following stroke.** Model 1: crude, no adjustment; Model 2: adjusted for age (continuous), sex (dichotomous), and educational years (continuous), Model 3: propensity score adjusted models accounting for confounding factors. Propensity scores were calculated from logistic regression models including age (continuous), sex (dichotomous), education (continuous), ever smoking (dichotomous), habitual alcohol consumption (dichotomous), severe disease (dichotomous), previous stroke or transitory ischemic attack (dichotomous), cardiovascular diseases (dichotomous), and other organic brain diseases (dichotomous), with NMDAR1-abs serostatus as dependent variable. OR: Odd’s ratio; 95% CI: 95% confidential interval; FU: follow-up.

Supplementary Table 6

|  | 12-months FU | | | | | | | | |
| --- | --- | --- | --- | --- | --- | --- | --- | --- | --- |
|  | Model 1 | | | Model 2 | | | Model 3 | | |
|  | **OR** | **95% CI** | **p-value** | **OR** | **95% CI** | **p-value** | **OR** | **95% CI** | **p-value** |
| global | **3.1** | 0.82 – 11.54 | 0.09 | **2.9** | 0.77 – 11.48 | 0.12 | **2.8** | 0.74 – 11.06 | 0.13 |
| language | **0.8** | 0.09 – 5.84 | 0.78 | **0.8** | 0.10 - 6.62 | 0.86 | **0.7** | 0.09 – 5.81 | 0.77 |
| memory | **4.8** | 1.73 – 13.12 | 0.01 | **3.7** | 1.28 – 10.41 | 0.02 | **3.8** | 1.33 – 10.82 | 0.01 |
| visuo-spatial | **2.2** | 1.05 – 4.75 | 0.04 | **2.0** | 0.92 – 4.48 | 0.08 | **2.0** | 0.95 – 4.43 | 0.07 |
| executive | **1.7** | 0.49 – 6.12 | 0.39 | **1.7** | 0.47 – 6.17 | 0.42 | **1.9** | 0.53 – 7.17 | 0.32 |
| attentional | **0.4** | 0.06 – 3.36 | 0.43 | **0.4** | 0.05 – 2.82 | 0.33 | **0.3** | 0.04 – 2.70 | 0.31 |

**Supplementary Table 6:** **Effect estimates of anti-NMDA-receptor GluN1 antibody serostatus on cognitive impairment from stepwise adjusted logistic regression models at 12-months following stroke.** Model 1: crude, no adjustment; Model 2: adjusted for age (continuous), sex (dichotomous), and educational years (continuous), Model 3: propensity score adjusted models accounting for confounding factors. Propensity scores were calculated from logistic regression models including age (continuous), sex (dichotomous), education (continuous), ever smoking (dichotomous), habitual alcohol consumption (dichotomous), severe disease (dichotomous), previous stroke or transitory ischemic attack (dichotomous), cardiovascular diseases (dichotomous), and other organic brain diseases (dichotomous), with NMDAR1-abs serostatus as dependent variable. OR: Odd’s ratio; 95% CI: 95% confidential interval; FU: follow-up.

Supplementary Table 7

| **6 – 12 months FU** | | | | | | | | | |
| --- | --- | --- | --- | --- | --- | --- | --- | --- | --- |
|  | Model 1 | | | Model 2 | | | Model 3 | | |
|  | **OR** | **95% CI** | **p-value** | **OR** | **95% CI** | **p-value** | **OR** | **95% CI** | **p-value** |
| global | **2.35** | 0.74 – 7.53 | 0.15 | **2.19** | 0.70 – 6.85 | 0.18 | **2.11** | 0.65 – 6.88 | 0.22 |
| language | **1.02** | 0.22 – 4.80 | 0.98 | **1.14** | 0.25 – 5.16 | 0.87 | **0.98** | 0.21 – 4.52 | 0.98 |
| memory | **2.99** | 1.33 – 6.73 | 0.01 | **2.59** | 1.18 – 5.72 | 0.02 | **2.41** | 1.05 – 5.49 | 0.04 |
| visuo-spatial | **2.13** | 1.10 – 4.11 | 0.03 | **1.96** | 0.96 – 4.01 | 0.07 | **1.84** | 0.92 – 3.69 | 0.09 |
| executive | **1.28** | 0.46 – 3.53 | 0.64 | **1.23** | 0.45 – 3.38 | 0.67 | **1.29** | 0.48 – 3.57 | 0.62 |
| attention | **0.98** | 0.41 – 2.36 | 0.96 | **0.94** | 0.38 – 2.35 | 0.90 | **0.83** | 0.33 – 2.08 | 0.69 |

**Supplementary Table 7: Effect estimates of anti-NMDA-receptor GluN1 antibody serostatus and cognitive impairment from stepwise adjusted logistic GEE models from 6 to 12 months follow-up.** Model 1: crude, no adjustment; Model 2: adjusted for age (continuous), sex (dichotomous), and educational years (continuous), Model 3: propensity score adjusted models accounting for confounding factors. Propensity scores were calculated from logistic regression models including age (continuous), sex (dichotomous), education (continuous), ever smoking (dichotomous), habitual alcohol consumption (dichotomous), severe disease (dichotomous), previous stroke or transitory ischemic attack (dichotomous), cardiovascular diseases (dichotomous), and other organic brain diseases (dichotomous), with NMDAR1-abs serostatus as dependent variable. OR: Odd’s ratio; 95% CI: 95% confidential interval; FU: follow-up.

Supplementary Table 8

|  | 6-months FU | | | 12-months FU | | |
| --- | --- | --- | --- | --- | --- | --- |
| Neuropsychological test, total score | DEMDAS  mean, SD | seronegative  mean, SD | seropositive  mean, SD | DEMDAS  mean, SD | seronegative  mean, SD | seropositive  mean, SD |
| CES-D | 12 (8) | 12 (8) | 12 (8) | 11 (8) | 11 (8) | 9 (7) |
| Fatigue score | 18 (13) | 18 (13) | 17 (13) | 16 (12) | 17 (12) | 14 (10) |

**Supplementary Table 8: CES-D and Fatigue Scores at 6- and 12-months follow-up stratified upon anti-NMDA-receptor GluN1 antibody serostatus**. CES-D: Center for Epidemiologic studies – depression scale, FU: follow-up.
